# Development of the Lithuanian Version of Sniffin’ Sticks 12 Odor Identification Test

**DOI:** 10.3390/medicina54020013

**Published:** 2018-04-11

**Authors:** Jolita Čičelienė, Žygimantas Vaičys, Daiva Rastenytė

**Affiliations:** Department of Neurology, Medical Academy, Lithuanian University of Health Sciences, 44307 Kaunas, Lithuania; zygimantas.vaicys@gmail.com (Ž.V.); daiva.rastenyte@lsmuni.lt (D.R.)

**Keywords:** smell, validation studies, olfaction disorders, healthy volunteers, Lithuania

## Abstract

*Background*: Evaluation of smell function is essential especially in cases of gradual deterioration, e.g., in neurodegenerative diseases, where rates of unawareness of the disorder are high and the importance of screening for olfactory dysfunction is increasing. To date, none of the tests for evaluation of olfactory dysfunction has been validated in Lithuania. The aim of the study was to develop a Lithuanian version of Sniffin’ Sticks 12 (SS12) odor identification test. *Materials and Methods*: The study was performed in 4 stages. The first stage included translation and back-translation from German, pilot group testing and language adaptation of the original SS12 test. In the second stage a survey group of 99 subjects was questioned for familiarity with the descriptors, used in the original version of the test. In the third stage after replacement of the least familiar distracters, a modified version of SS12 was created. Original and modified versions of SS12 were tested on 112 and 119 healthy subjects accordingly. The fourth stage of the study proved necessary as neither of the two SS12 versions turned out to be valid. After another round of replacement of the misleading distracters the second modified version of SS12 was created and it was tested on 115 healthy subjects. *Results*: Unsatisfactory correct identification rates of less than 75 percent in the same one item (lemon) were observed using both original and modified SS12 versions. With the second modification of distracters of SS12, identification of lemon increased significantly and overcame 75 percent. The decrease of SS12 scores in relation to age was ascertained in the study sample. Gender and smoking status did not prove to be independent predictors of SS12 scores in multiple linear regression analysis. *Conclusion*: The study presents an olfactory testing tool, which is adapted and modified culturally for use in the Lithuanian population.

## 1. Introduction

The sense of smell is one of the essential evolutionary defense mechanisms in the human body. The intact olfactory system ensures discerning spoiled food and detecting some poisonous gases in the environment, thus enabling the avoidance of certain health threats. Furthermore, intact olfactory function plays a role in savoring food, experiencing different emotions and overall quality of life [1]. However, frequently the subconscious nature of the sense makes it difficult to realize the deficit of the smell function. Studies in different populations show marked unawareness of olfactory impairment by hyposmic subjects [2,3], especially when there is no acute phase of deterioration in olfactory performance, as seen in cases where olfactory deficit is acquired after acute respiratory tract infections. Thus, standardized measures are needed to determine whether a certain patient has any impairment of smell sensation. The interest in detecting smell impairment is not limited to the scope of a rhinolaryngologist. Evidence is emerging about the role of detection of olfactory impairment together with other possible predictors in screening for neurodegeneration [4,5].

The diagnostic tools for routine clinical investigations must be as simple, as quickly performed, and as inexpensive as possible. One of the widely used smell tests is Sniffin’ Sticks battery [6] with its short versions for smell identification [7]. Currently the manufacturer is offering three versions of the Sniffin’ Sticks smell identification test—two different sets of 16 odors (SS16—the blue and the purple one) and a set of 12 odors (SS12). The whole battery, or at least one of the identification part versions of Sniffin’ Sticks, has been adapted and validated for use in different European countries, such as Great Britain [8], the Netherlands [9], Romania [10], Italy [11], Portugal [12], Denmark [13], Estonia [14], Poland [15], Greece [16,17], Turkey [18,19], and Switzerland [20] as well as in other continents (Taiwan [21], Sri Lanka [22], Egypt [23], Mexico [24], and Australia [25]).

Familiarity with odors depends largely on the frequency of encounter with any specific odor. Prevalence of certain plants in the local area, peculiarities of local cuisine, and gourmet culture of the nation, among other factors, can influence some test options to be unrecognizable by the inhabitants of different countries. Therefore, the adaptation of the Sniffin’ Sticks test should not be limited to literal translation of the default options, but it should be tested for suitability, and in some cases substitution for misleading distracters is preferable.

To date, none of the tests for evaluation of olfactory function has been validated for use in the Lithuanian population. We chose to adopt SS12—the identification part of the more complex olfactory testing set Sniffin’ Sticks. It includes 12 pen-sticks containing different odors that patients are expected to be familiar with and an answer sheet with a correct descriptor and 3 distracters for each odor. The aim of the study was to create culturally suitable version of SS12 and to suggest its normative values. With the further interest in using the test for neurodegenerative diseases, the focus of the study was not limited to the younger population. We included adult participants of all age groups to additionally obtain the evaluative data for older patients with no known neurodegeneration, considering the reported decreased olfactory function with age [7,10,11,12,19,23,24,25].

## 2. Materials and Methods

### 2.1. Participants and Ethical Considerations

Study participants were recruited from the patients, their relatives and personnel of the Departments of Neurology and Cardiology in Lithuanian University of Health Sciences Hospital, Kauno Klinikos. The study enrolled 455 subjects. Demographic data were collected from the participants regarding age, gender, and smoking habits.

The study protocol met the criteria of the World Medical Association Declaration of Helsinki and was approved by Kaunas Regional Biomedical Research Ethics Committee (No. BE-2-70, issued on 5 November 2010).

**Inclusion criteria**. Native Lithuanian speakers over 18 years of age who gave the informed consent were enrolled in the study if they did not meet the exclusion criteria.

**Exclusion criteria**. The exclusion criteria were self-reported or documented comorbidities or conditions that may have influenced the olfactory performance of the participants. During the structured interview the potential participants were asked if they felt they had any kind of smell dysfunction of any duration, or nasal congestion symptoms on the day of testing. Disorders of central nervous system, including neurodegenerative diseases as well as history of major head or nose traumas were ruled out using the structured interview and inspection of the medical records where available. Mini-mental State Examination (MMSE) was performed to otherwise eligible participants to rule out possible signs of cognitive impairment. Subjects who scored less than 24 points were not enrolled in the study.

Assuming the prevalence of olfactory impairments to be at least 22% in the general population [2,26], the study would require a sample size of 103 subjects for estimating the expected proportion with 8% absolute precision and 95% confidence [27]. The inclusion of participants who were tested with different modifications of SS12 was balanced for equal distribution of gender and in all decades of adult age. Study participants were categorized for further statistical analysis into the following age groups: A: 18–40 years, B: 41–60 years, C: >60 years.

### 2.2. Study Procedures

**Figure 1:**
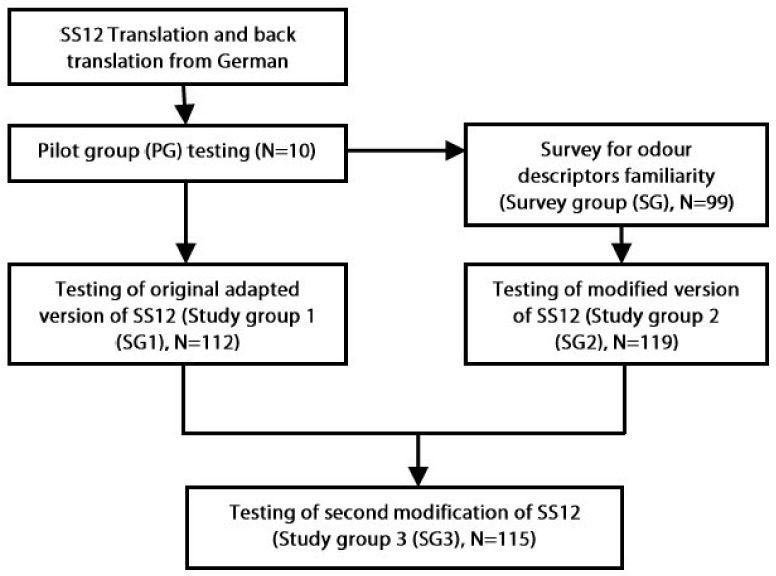
Flowchart of study procedures. SS12—Sniffin’ Sticks 12.

The study was performed in stages, which are summarized in Figure 1 and described in more detail below.

**Smell testing**. For testing of olfactory function, the identification part of the Sniffin’ Sticks battery [6]—SS12 [7] was used. Subjects were given each of 12 pen-sticks to smell and to choose one of the 4 suggested answers, which would describe the given odor in the best way. For odor presentation the cap of the pen was removed for 3–4 s and positioned approximately 2 cm in front of both nostrils of the participant and he was asked to sniff. Participants could repeatedly smell the pen if they were unsure. An interval of at least 30 s between pens was kept. The subjects were asked to pick one descriptor even if they did not smell anything, or they were not sure about the correct answer, in latter cases—by rejection of the more unlikely descriptors. The result of the test was the score of correct answers given.

**Language adaption of SS12**. The original SS12 test was translated to Lithuanian and back-translated to German. To evaluate the comprehension of the test options, the Lithuanian version of the original test was applied to a pilot group. Participants were asked to choose one odor descriptor from a list of four for each pen-stick and to provide comments or suggestions of alternative descriptors if they had any. Minimal corrections to the original translation were made according to these suggestions of the pilot group and the adapted original version of SS12 was created for Lithuanian language.

**Survey for familiarity with original SS12 descriptors in Lithuania**. Participants of the survey group were inquired for familiarity with all the descriptors of the adapted original SS12 test. Without having to actually smell the pen-sticks, they were asked whether they thought they would recognize each odor (yes/no). Furthermore, as implemented by Antsov et al. [14], these subjects were asked to suggest more odors with distinctive fragrant qualities that they would be familiar to.

**Modification of SS12**. After assessing the results of the odor survey, the most unfamiliar distracters used in the test (those indicated as unfamiliar by >10 percent of the survey group subjects) were changed to more familiar ones. These were selected from a list of additional odors suggested by at least five independent subjects from the survey group and judged as the most appropriate alternative distracters by the investigators. In cases where the correct test option was indicated as unfamiliar by more than 10 percent of the survey participants, the distracters were modified so that the correct answer could be chosen by rejection. Based on these changes of distracters, a modified version of SS12 test was developed.

**Testing of the original adapted and modified versions of SS12**. The authors of the test have set the prerequisite of successful identification rate of each odorant to be at least 75 percent of healthy subjects [6]. Original adapted and modified versions of SS12 were tested on two different groups of subjects. After reviewing the results from the two groups, it was observed that there are items in both test versions which do not reach the 75 percent of correct identification rates. Therefore, another modification of the most problematic descriptors was made. This time the alternate descriptors were chosen from those already in the test. The selected descriptors were used repeatedly as distracters for different odors. Odorants which in the investigators’ opinions are most commonly encountered in everyday life of average Lithuanian person were chosen for repeated use. The second modified version of the SS12 was tested on another group of participants.

### 2.3. Statistics

Statistical analysis was performed using SPSS v20 (IBM Corp., Armonk, NY, USA). Data is presented as mean ± standard deviation (SD) when Gaussian distribution of the continuous data was observed, and as median (Interquartile range) when the distribution was not normal. The categorical data are presented as percentages. Chi-square with post-hoc Bonferroni test was used to evaluate the differences among the proportions of correct answers in SS12 items as well as gender and smoking status distribution among SS12 modifications. Kruskal-Wallis with post-hoc Bonferroni test was used to evaluate differences in age and SS12 results among SS12 modifications as well as differences in SS12 among age groups. Multiple linear regression analysis was performed to evaluate the factors that independently influenced the results of SS12. SS12 score was used as a dependent variable and age, gender and smoking status were used as covariates. The level of significance was set at 0.05. For identification of cut-off values for hyposmia 10th percentile of correct SS12 score was used. For identification of cut-off values for anosmia the statistical probability modelling was used. The probability of obtaining a certain number of correct answers by chance was calculated using the binomial distribution Equation (1) [28].

f(X = k/n) = ((n!/k!(n − k)!) × pk × qn − k (*n* = 12, p = 0.25, q = 0.75) (1)

## 3. Results

Distribution of gender, age, and smoking habits did not differ across the three study groups (SG1, SG2 and SG3) (*p* > 0.05).

### 3.1. Pilot Group Testing and Adaption of Original SS12

The pilot group consisted of 10 subjects aged 23–46 years (31.8 ± 7.8), 4 of whom were men. After testing the pilot group, it was noticed that the option licorice was mostly unfamiliar to the tested subjects. Part of them (6/10) indicated that it smelled like anise, which was not among the test options: two of the six did not select any of the suggested answers, the remaining four chose licorice by rejection. Presuming that licorice is mostly unfamiliar to the Lithuanian population (which proved to be true in the next step of surveying for familiarity of the odors) and that licorice and anise, despite unrelated origin, share some fragrant qualities, we supplemented the correct option licorice to licorice/anise.

### 3.2. Survey Group Testing and Modification of SS12

The survey group consisted of 99 subjects, aged 18–85 years (59.71 ± 16.41) of whom 44 (44.44%) were men. Of the suggested 44 descriptors, 14 were rated as unfamiliar by more than 10 percent of the odor familiarity survey participants (Table 1). Of those, four belonged to the proposed correct answers of the SS12, therefore, were considered irreplaceable. The rest of 10 unfamiliar distracters (one of them (peach) mentioned in the answer sheet twice) were replaced by more familiar ones, selected from the options additionally suggested by the survey group. In cases of unfamiliar correct options, two additional distracters (grass and gummy bear) were replaced to make it easier to choose the correct unfamiliar answer by rejection. The steps of modification of distracters used in SS12 are presented in Table 2.

### 3.3. SS12 Results in Three Study Groups

The adapted original version of SS12 was tested in 112 SG1 participants aged 22–88 years (51.64 ± 18.39), of whom 52 (46.4 percent) were men, 23 (20.5 percent) current smokers and 20 (17.9 percent) former smokers. The distribution of participants in the three age groups was as follows: A—34, B—36, C—42 participants. The modified version of SS12 was tested in 119 SG2 participants aged 23–89 years (53.74 ± 18.76), of whom 53 (44.5 percent) were men, 15 (12.6 percent) current smokers and 17 (14.3 percent) former smokers. The distribution of participants in the three age groups was as follows: A—34, B—35, C—50 participants. The second modified version of SS12 was tested in 115 of SG3 participants, aged 18–88 years (51.77 ± 18.37), of whom 53 (46.1 percent) were men, 23 (20 percent) current smokers and 19 (16.5 percent) former smokers. The distribution of participants in the three age groups was as follows: A—34, B—35, C—46 participants.

The correct identification rate of each odorant with different modifications of the answer sheet is presented in Table 3. The first modification of SS12 answer sheet significantly improved problematic identification of lemon observed in the original adapted version but did not reach 75 percent. Therefore, with another modification of seven distracters observed to cause most of the confusion, a third version of SS12 answer sheet was created. The list of final distracters as well as the intermediate steps of their choice are shown in Table 2. The identification of lemon improved significantly in the first modification, but the identification rate was still unsatisfactory as it did not reach 75 percent. In the second modification the identification of lemon improved significantly compared to both former versions of the test and overcame the prerequisite of 75 percent. The identification of pineapple improved significantly in the second modification compared to the first modification (Table 3). Identification of other descriptors did not change significantly. The median SS12 result did not change significantly with any modification and equaled 10 (Interquartile range (IQR) 9–11) for SG1, 11 (IQR 10–11) for SG2 and 11 (9–12) for SG3 (*p* > 0.05). The validated Lithuanian test version is depicted in Table 4.

For further statistical analysis only the results of SG3 were used. A significant difference of SS12 scores among 3 age groups was observed (*p* < 0.001) (Table 5). The post hoc analysis revealed the distribution of SS12 in group C to differ from A (*p* < 0.001) and B (*p* = 0.011), with no significant difference in the latter two. After testing the assumptions, multiple linear regression analysis was performed to predict SS12 scores from age, gender and current as well as past smoking status. Only age (*r* = −0.037, *p* < 0.001) proved to be significant independent predictor of SS12 score, F (4.110) = 9.052, *p* < 0.001. *R*^2^ = 0.248, meaning that the model explains 24.8 percent of SS12 score variation.

The 10th percentile of SS12 correct identification rates in the reference age group (18–40 years) was 9.5. This value should be treated as a cut-off value between normosmia and hyposmia, thus correct SS12 identification rates through 9 downwards should be treated as impaired olfactory function despite subject’s age. The 10th percentile of SS12 identification rates in older age groups is presented in Table 5.

**Figure 2:**
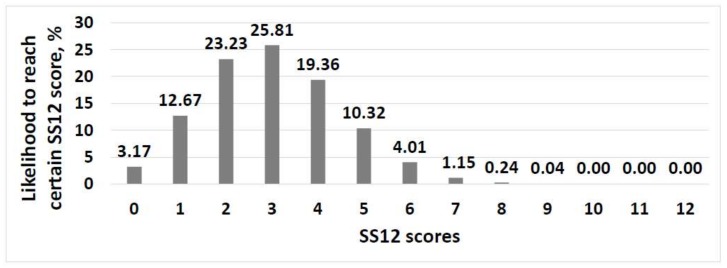
Computed distribution of likelihood to reach certain SS12 scores, assuming random performance. SS12—Sniffin’ Sticks 12.

The cut-off value for anosmia was set based on the statistical probability modelling. The distribution of likelihood to reach certain correct SS12 identification rates are presented in Figure 2. We suggest scores with random performance probability over 4 percent (i.e., 1–6) to be regarded as indicative of anosmia.

## 4. Discussion

To our knowledge this is the first study to adapt a tool for quantitative evaluation of the olfactory function in Lithuania. The result of the study is an olfactory testing tool which is adapted and modified culturally for Lithuanian population. The normative values are suggested for assessment of olfactory function.

During the modification process of SS12 the most problematic item in our study surprisingly appeared to be lemon, which was not named as unfamiliar by any of the survey participants, but disproportionally large part of subjects in SG1 and SG2 could not recognize this odor. The same problem was observed in some other studies [8,10,13,14,17]. The possible flaw may be a distracter with similar citric fragrant qualities—grapefruit, which in our case was chosen instead of lemon by 33.9 percent of the SG1 subjects. Nevertheless, after modification of distracters for this odor, the correct identification rate, despite increasing significantly, did not reach the prerequisite of 75 percent. This time nearly quarter of SG2 subjects chose the modified distracter jasmine. We therefore hypothesize that either the odor of lemon may differ in those imported fruits that reach Lithuania, with the climate not suitable to grow our own lemons or that we are prone to attribute misleading synthetic scent of “lemon” used in most of the household chemicals to the odor of the fruit itself which may be used less often. Similar problem with the odor itself was noted in Turkish study [19] where apple was not well recognized by healthy subjects. The proposed explanation was that the locally cultivated apples tend to have different fragrant qualities and the odor was reminiscent of the popular air freshener rather than a fruit. In such cases, when the perceived odor differs from the expected, the choice of the descriptor becomes based on rejection with the choice of the least familiar one. In our case introducing jasmine as a distracter proved to be a mistake. Even though it was suggested by eleven independent subjects from the survey group as a well-known odor, their choice of jasmine may have been conditioned by its distinct and potent fragrant qualities but not necessarily by its widespread familiarity. Similarly, lily of the valley (suggested by 5 independent subjects), which was chosen to substitute plum in the distracters for pineapple, was mistakenly chosen by 11.5 percent of the SG2 subjects.

As such systematic errors may reduce the specificity of the test, another modification of the descriptors for validation of Lithuanian version of SS12 proved necessary. Second modification of Sniffin’ sticks identification subscale also had to be performed in the development of Arabic version of the test [23]. Gudziol et al. have found that the results of odor identification test (Sniffin’ Sticks 16) differ significantly when different distracters are used for each item [29]. The main source of the difference was observed to be the improved performance of hyposmic subjects with more contrast distracters [29]. The authors suggest that better discrimination between anosmic and hyposmic subjects which could be achieved by implementing more contrast distracters is highly valuable in clinical context [29]. With the goal to raise the recognizability of the problematic items, our choice of the distracters for second modification of SS12 was based on the criteria of evident difference from the correct descriptor and widespread encounter in daily life of subjects of any age. With the results of the first modification of SS12 in mind, we reconsidered against introducing new untested distracters instead of the problematic ones and rather choose from the ones already in use in the original test. Based on these modifications, the third version of SS12 proved to overcome the prerequisite of 75 percent correct identification rate for every single item. Even though the identification rates of a few items lowered in the final version of SS12, the differences were not statistically significant. Neither of distracters was changed in the second modification of SS12 for two of the descriptors that went down—cinnamon and coffee. These changes were attributed to natural variation. Cronbach’s alpha criterion was not used to test for the internal validity as the test choices are non-ordinal categorical variables. The modified SS12 version is valid for use in the Lithuanian population.

Normative values in the original development of SS12 were based on the 90th percentile of the hyposmic individuals with the observation that there may be a significant overlap between the scores of normosmics and hyposmics as well as between hyposmics and anosmics [7]. The authors suggest further testing for subjects, who score in the range of the possible overlap between hyposmia and anosmia, thus suspecting them hyposmic based on the screening test. In contrast, the suggested cut-off values between normosmia and hyposmia in most of the Sniffin’ Sticks validation studies, are set at the 10th percentile of the young normosmic adults [16,19,21,23,24,25,30,31]. In cases when the intended use of the test is for screening purposes, the false positive result of hyposmia is undesirable and may result in further unnecessary testing. We suggest the cut-off value for normosmia to be set at the 10th percentile of the participants younger than 40 years, treating scores through 9 downwards as hyposmic.

In multiple linear regression analysis, age proved to be the one independent predictor of SS12 scores. The decline in correct SS12 scores with age was similarly reported by other groups [7,10,11,12,19,23,24,25,30]. In certain situations, differentiation of smell impairments from senile hyposmia may be needed (e.g., in neurodegenerative diseases). Therefore, the reference data from older age groups may be of value and, therefore, 10th percentile values of the SS12 observed in the older age groups are presented in the results.

It was confirmed by Kobal et al. that the performance of anosmics in Sniffin’ Sticks testing battery as well as the identification part of the test (SS16) conformed the probability of random performance [30]. Therefore, without having objective measures to confirm anosmia, we calculated probabilities of random performance in SS12 variant of the test. We suggest slightly modifying one percent probability cut-off values for indicating anosmia as proposed by Kobal et al. [31] or five percent cut-off values suggested by Wolfensberger M et al. [20]. Both of the above-mentioned studies used the whole Sniffin’ Sticks battery, including SS16, but not SS12 as in our study. Therefore, in our case, the probabilities differ slightly. The authors of the original SS12 modification [6] have found the 90th percentile of anosmics to be at the score of 6 in SS12. The probability of such score, if assuming random performance, is slightly over 4 percent. In contrast, the probability of the score of zero is slightly less than 4 percent. Using one percent likelihood as a cut-off value in SS12 would include the score of 0 in the “anosmic” range of SS12 results. The authors of the test argue against such rating in the test instructions (Burghart Messtechnik GmbH. Sniffin’ Sticks Screening 12 Test, Instructions for Use, 2012), suggesting that a zero score could be a sign that the subject gives wrong answers on purpose. Therefore, we suggest using a cut-off value of 4 percent of random performance probability for anosmia. This makes a score of 7 or more very unlikely for the anosmic patient as well as the score 0—which may be considered malingering.

The suggested cut-off values are to be taken into consideration cautiously because of the small study sample, lack of objective measuring of olfactory function and no patient group with impaired smell function in the study. Therefore, these should not be used in medico-legal context or strict clinical categorization of patients. However, they could serve as a preliminary reference values for further validation in larger cohort studies.

As validation studies differ in design, statistics, age groups, size of the studied population and number of items in the SS identification test, it would be incorrect to make direct comparisons of the olfactory abilities of our population with the populations in other countries.

## 5. Conclusions

The study presents an olfactory testing tool which is adapted and modified culturally for use in the Lithuanian population. Further cohort studies are needed to validate the suggested cut-off values of the test scores.

## Figures and Tables

**Table 1:** Most unfamiliar items of adapted original SS12 version.

Descriptor	Unfamiliarity Rate, %	Descriptor	Unfamiliarity Rate, %	Descriptor	Unfamiliarity Rate, %
Licorice *	76	Spearmint	20	Plum	14
Blackberry	35	Leather *	19	Mustard	13
Peppermint *	33	Grapefruit	19	Anise *	13
Coconut	28	Peach	17	Glue	12
Walnut	28	Cookies	14		

* Correct descriptors in the proposed SS12 test answer sheet. Items, indicated as unfamiliar by more than 10 percent of Survey Group (SG) subjects (*n* = 99) were considered unfamiliar. SS12—Sniffin’ Sticks 12.

**Table 2:** The distracters for each correct descriptor and their changes in modifications of SS12.

Descriptor	Distracters		
Orange	Blackberry → Lilac	Strawberry	Pineapple
Leather	Glue → Gasoline	Grass → Hay	Smoke ⇒ Beer
Cinnamon	Vanilla	Chocolate	Honey
Peppermint	Chive	Fir	Onion
Banana	Coconut → Garden strawberry	Walnut → Cucumber	Cherry ⇒ Cheese
Lemon	Peach → Jasmine ⇒ Ham	Grapefruit → Chocolate	Apple
Licorice/Anise	Gummy bear → Thyme ⇒ Orange	Spearmint → Mint	Cookies → Beer
Coffee	Cigarette	Wine	Smoke
Clove	Mustard → Parsley	Pepper ⇒ Garlic	Cinnamon
Pineapple	Plum → Lilly of the valley ⇒ Onion	Peach → Linden tree	Pear
Rose	Chamomile ⇒ Chocolate	Raspberry	Cherry
Fish	Bread	Cheese	Ham

The distracters substituted in the first modification follow the sign “→”. The distracters substituted in the second modification follow the sign “⇒”. SS12—Sniffin’ Sticks 12.

**Table 3:** Correct odor identification rates with SS12 answer sheet modifications.

Correct Descriptor	Original Adapted Version, % of Correct Identification in SG1 (*n* = 112)	Modified Version, % of Correct Identification in SG2 (*n* = 119)	Second Modified Version, % of Correct Identification in SG3 (*n* = 115)	*p*
Orange	97.3	96.6	99.1	>0.05
Leather	75.0	78.2	85.2	>0.05
Cinnamon	82.1	83.2	76.5	>0.05
Peppermint	87.5	92.4	92.2	>0.05
Banana	83.9	81.5	77.4	>0.05
Lemon	51.8 **	73.1 **	86.1 **	<0.001
Licorice/Anise	84.8	87.4	80.0	>0.05
Coffee	95.5	91.6	87.0	>0.05
Clove	82.1	83.2	87.0	>0.05
Pineapple	79.5	77.3 *	89.6 *	0.034
Rose	85.7	81.5	91.3	>0.05
Fish	94.6	93.3	95.7	>0.05

* Significant at the 0.05 probability level, ** Significant at the 0.01 probability level. SS12—Sniffin’ Sticks 12.

**Table 4:** Validated Lithuanian version of SS12.

1	Apelsinas *	Alyvos	Žemuogė	Ananasas
2	Alus	Benzinas	Galanterinė oda *	Šienas
3	Medus	Vanilė	Šokoladas	Cinamonas *
4	Česnakas	Pipirmėtė *	Eglė	Svogūnas
5	Braškė	Bananas *	Agurkas	Sūris
6	Kumpis	Obuolys	Citrina *	Šokoladas
7	Saldymedis/Anyžius *	Apelsinas	Mėta	Alus
8	Cigaretė	Kava *	Vynas	Dūmai
9	Gvazdikėliai *	Pipirai	Cinamonas	Petražolė
10	Kriaušė	Svogūnas	Liepa	Ananasas *
11	Šokoladas	Avietė	Rožė *	Vyšnia
12	Duona	Žuvis *	Sūris	Kumpis

* Correct descriptors. SS12—Sniffin’ Sticks 12.

**Table 5:** Distribution of correct odor identification (SS12) rates by age groups.

	Age Group A (18–40)	Age Group B (41–60)	Age Group C (>60)
Number of subjects	34	38	43
Median of SS12 score	12	11	10 *
Interquartile range of SS12 score	10.75–12	10–12	9–11
10th percentile of SS12 score	9.5	9	7.4

* Significant at the 0.05 probability level. SS12—Sniffin’ Sticks 12.
